# Aggressive Rhythm Control Strategy in Atrial Fibrillation Patients Presenting at the Emergency Department: The HEROMEDICUS Study Design and Initial Results

**DOI:** 10.3390/jcdd11040109

**Published:** 2024-03-31

**Authors:** Dimitrios Tsiachris, Nikos Argyriou, Panagiotis Tsioufis, Christos Konstantinos Antoniou, Aggeliki Laina, George Oikonomou, Ioannis Doundoulakis, Athanasios Kordalis, Kyriakos Dimitriadis, Konstantinos Gatzoulis, Konstantinos Tsioufis

**Affiliations:** First Department of Cardiology, School of Medicine, Hippokration General Hospital, National and Kapodistrian University of Athens, 11527 Athens, Greece; nikos_ar@hotmail.com (N.A.); ptsioufis@gmail.com (P.T.); ckantoniou@hotmail.gr (C.K.A.); agelikilaina@hotmail.com (A.L.); geooik88@gmail.com (G.O.); akordalis@gmail.com (A.K.); dimitriadiskyr@yahoo.gr (K.D.); kgatzoul@med.uoa.gr (K.G.); ktsioufis@gmail.com (K.T.)

**Keywords:** atrial fibrillation, emergency department, cardioversion, flecainide, electrical cardioversion

## Abstract

Atrial fibrillation has progressively become a more common reason for emergency department visits, representing 0.5% of presenting reasons. Registry data have indicated that about 60% of atrial fibrillation patients who present to the emergency department are admitted, emphasizing the need for more efficient management of atrial fibrillation in the acute phase. Management of atrial fibrillation in the setting of the emergency department varies between countries and healthcare systems. The most plausible reason to justify a conservative rather than an aggressive strategy in the management of atrial fibrillation is the absence of specific guidelines from diverse societies. Several trials of atrial fibrillation treatment strategies, including cardioversion, have demonstrated that atrial fibrillation in the emergency department can be treated safely and effectively, avoiding admission. In the present study, we present the epidemiology and characteristics of atrial fibrillation patients presenting to the emergency department, as well as the impact of diverse management strategies on atrial-fibrillation-related hospital admissions. Lastly, the design and initial data of the HEROMEDICUS protocol will be presented, which constitutes an electrophysiology-based aggressive rhythm control strategy in patients with atrial fibrillation in the emergency department setting.

## 1. Introduction

Atrial fibrillation (AF) is the most common sustained cardiac arrhythmia in the adult population globally, with an estimated prevalence between 2% and 4% [1]. Both the incidence and prevalence of AF are expected to increase further due to population aging and the increasing burden of other comorbidities, such as hypertension, heart failure, obesity, diabetes mellitus, coronary artery disease, and chronic kidney disease [2,3,4,5,6,7]. AF is associated with increased morbidity [8], mortality [9], and higher healthcare costs, rendering it a major socioeconomic burden [10]. AF has progressively become a more common reason for emergency department (ED) visits, representing 0.5% of presenting reasons [11].

Registry data have indicated that about 60% of AF patients who present to the ED are admitted, leading to an increase in AF-related hospital admissions due to the aforementioned rise in ED visits, emphasizing the need for more efficient management of AF in the acute phase [11,12,13,14]. Several trials of AF treatment strategies, including cardioversion, have demonstrated that AF in ED can be treated safely and effectively, avoiding admission [11,12,13,14,15,16,17,18,19,20,21,22,23,24,25,26].

In the present study, the epidemiology and characteristics of AF patients presenting to the ED will be reviewed, as well as the impact of diverse management strategies on AF-related hospital admissions. Lastly, the design and initial data of the HEROMEDICUS protocol will be presented, which constitutes an electrophysiology-based aggressive rhythm control strategy in patients with AF in the ED setting.

## 2. AF-Related Visits to the ED

Initial data were published by McDonald et al. in 2008 drawn from the National Hospital Ambulatory Medical Care in the United States [14]. In the time period from 1993 to 2004, the absolute number of visits increased from 300,000 (1993-4) to 564,000 (2003-4). Likewise, the population-adjusted visit rate increased from 0.6 to 1.2 per 1000 members of the US population [14]. Accordingly, data from the Analysis of the Nationwide Emergency Department Sample revealed that ED visits for AF and atrial flutter (AFL) increased from 434,382 in 2006 to 568,562 in 2011, corresponding to a 30.9% increase over 6 years [13]. More specifically, ED visits per 1000 members of the population increased from 1.45 in 2006 to 1.82 in 2011 [13]. Subsequent data from a repeated cross-sectional analysis of the ED visit level from the Nationwide Emergency Department Sample confirmed the increase was largely sustained (at 30.7%) for annual ED visits for AF from 2007 to 2014 [11] (Table 1).

## 3. AF Admission Rates in the ED

During the 12-year study period (1993–2004) of the National Hospital Ambulatory Medical Care Registry, the admission rate remained constant (64%) [14]. Concomitant congestive heart failure was associated with an increased risk of hospital admission [14]. Likewise, ED admission rates overall persisted at a high level (between 67.4% and 69.7%) in the 2006–2011 period, as derived from the Nationwide Emergency Department Sample database [13]. The hospital region and presence of comorbidities were the strongest predictors of admission in this study [13]. Subsequent data from the same database revealed stable hospital admission rates at ~70% between 2007 and 2010, after which they declined to 62% in 2014, despite an increase by 30.7% in annual ED visits [11] (Table 2).

## 4. AF Cost of Hospital Stay

The annual cost of AF treatment was estimated to be USD 6.65 billion in 2005 [10] and later estimates were even higher [22]. In 2007, inpatient charges for AF hospitalization accounted for 1.12% of USD 530 billion and, in 2014, this further increased to 1.22% of USD 828 billion [11]. There is a huge difference between the charges spent on hospitalized AF patients and charges spent on those discharged from the ED. In 2007, annual charges for AF hospitalization were estimated at USD 7.39 billion, increasing by 37% to USD 10.1 billion in 2014. Furthermore, per patient hospitalization charges increased from USD 17,317 in 2007 to USD 22,113 in 2014 [11].

A smaller European study examined the impact on hospital resources of two different approaches to recent-onset AF using a random sample of 300 patients with AF, collected from an ED electronic database from an urban community teaching hospital. The median charge for patients cardioverted and discharged from the ED was USD 5460 (IQR USD 4677–6190) whereas the median charge for admitted patients with no attempt at cardioversion at the ED was at least four times higher (USD 23,202, IQR USD 19,663–46,877) [16].

## 5. Strategies for AF Management in the ED

Management of AF comprises three main domains summarized in the “ABC” scheme of the 2020 European Society of Cardiology (ESC) AF guidelines: these are “A” for anticoagulation/avoid stroke, “B” for better symptom control using rate and rhythm management, and “C” for therapy of concomitant cardiovascular conditions [27,28,29,30]. The current use of rhythm control therapy is based on evidence generated in key trials originally conducted to compare rhythm vs. rate control strategies. These include the following studies: PIAF (Pharmacological Intervention in Atrial Fibrillation), AFFIRM (Atrial Fibrillation Follow-up Investigation of Rhythm Management), RACE (Rate Control Versus Electrical Cardioversion for Persistent Atrial Fibrillation), AF-CHF (Atrial Fibrillation and Congestive Heart Failure), STAF (Strategies of Treatment of Atrial Fibrillation), and J-RHYTHM (Japanese Rhythm Management Trial for Atrial Fibrillation) [31,32,33,34,35,36]. A few significant differences have been reported in important endpoints between rhythm control and rate control strategies in the above-mentioned trials, and meta-analyses have indicated that fewer hospitalizations were required to deliver simple rate control [32,33,34]. As a consequence, initial treatment points towards rate control, while rhythm control is reserved to improve symptoms that persist despite adequate rate control.

In the multicenter, randomized EAST-AFNET 4 trial, a strategy of initiating rhythm control therapy in all patients with early AF and concomitant cardiovascular conditions was associated with a lower risk of death from cardiovascular causes, stroke, or hospitalization for heart failure or acute coronary syndrome than the usual care over a follow-up time of more than 5 years (absolute difference in risk was 1.1 events per 100 person-years) [37]. Early rhythm control did not affect the number of nights spent in the hospital [37]. The absence of an appreciable difference in hospital nights is reassuring in view of the excess hospitalizations associated with rhythm control therapy reported in the AFFIRM and AF-CHF trials [32,33,34].

There is no specific algorithm in the ESC guidelines on the management of hemodynamically stable patients presenting to the ED with primary AF, nor is there guidance on when to admit or discharge them straight from the ED [27,28,29,30]. There are multiple protocols that have examined the safety and efficacy of diverse strategies implemented in the ED for patients presenting with AF as the primary diagnosis. The basic goal of all different pathways is the reduction of hospital admissions (Table 3).

Burton et al. published data in 2004 on the feasibility of electrical cardioversion in the management of AF in the ED [18]. In this multicenter cohort study, the objective was to identify the outcomes and complications associated with electrical cardioversion in AF patients [18]. The study population included 388 patients with recent-onset AF (<48 h) and electrical cardioversion was successful in 332 patients (86%). In total, the admission rate was as low as 14% since 333 patients were discharged home from the ED (301 after successful cardioversion and 32 with electrical cardioversion failure). Interestingly, 39 patients returned to the ED because of AF recurrence within 10 days [18].

A multicenter, observational, cross-sectional study (HERMES-AF) of patients with recent-onset AF, which was carried out in 124 Spanish EDs from 23 May to 5 June 2011, studied the impact of a rhythm control strategy on admission rates and symptoms [24]. The inpatient admission rate was 14% in the rhythm control strategy. The study population totaled 421 patients, of which 352 were allocated to the rhythm control strategy while rate control was chosen in 69 patients. In total, 60 (14.2%) patients were admitted. Control of symptoms was achieved in 95.2% of patients in the rhythm and in 88.4% of patients in the rate control group [24].

In a prospective, two-stage study at two tertiary care hospitals in the northeastern United States, the utilization of a multidisciplinary AF pathway was associated with a decrease in hospital admission rates [25]. During the first stage of the study (27 June to 4 October 2016), AF patients who presented to the ED were treated according to the routine care. During the second stage of the study (5 October to 20 March 2017), patients who presented to the ED with primary AF were screened and treated according to the AF pathway [25]. Implementation of the AF treatment pathway was associated with decreased rates of hospital admission (15%) compared to the group who received routine care (55%) [25]. This was the first study that included an expert electrophysiologist besides emergency medicine doctors in decision making and AF management in the ED.

Canadian emergency physicians are known for publishing widely on the “AF in the ED” field and for managing these patients quickly and efficiently [20,23,26]. Academic centers in Canada have implemented a series of aggressive protocols of AF management in the ED setting [20,23,26]. An initial retrospective cohort study by Stiell et al. reported the results of pharmacological cardioversion with intravenous (i.v.) procainamide in 341 patients with recent-onset AF or atrial flutter. The sinus rhythm was restored in 52% of AF patients and 28% of atrial flutter patients [20]. Admission rates were only 5.6% and the adverse event rate was reported to be 10%, though without necessitating hospital admission. The return rate to the ED among those who were successfully cardioverted was 2.9% within 7 days [20]. Subsequently, electrical cardioversion was included in this procainamide-based protocol for those not successfully cardioverted with i.v. procainamide [26]. This modified protocol—called the “Ottawa aggressive protocol”—was evaluated in a retrospective cohort study. Sinus rate restoration was achieved in 92% of cases and the minor adverse event rate was 7.6% [26]. Certain steps included in the Ottawa aggressive protocol need to be mentioned. Specifically, no heparin or warfarin administration was necessary if it was clearly less than 48 h since the onset. Furthermore, importantly, procedural sedation and analgesia (i.v. propofol and fentanyl) was given by the emergency physicians without the use of transesophageal echocardiography unless the onset was unclear [20,26].

In the same direction, ED patients at six urban Canadian centers with uncomplicated AF of less than 48 h duration and a CHADS_2_ score of 0 or 1 were randomized in a 1:1 ratio to either chemical cardioversion with procainamide infusion, followed by electrical cardioversion if unsuccessful, or to electrical cardioversion followed by procainamide infusion if unsuccessful [23]. The primary endpoint was the proportion of patients being discharged within 4 h of arrival. The results of the study showed that both strategies appeared to be effective and well-tolerated but the electrical-first strategy was associated with less time spent in the ED [23].

All the above led to the development of the Acute AF/AFL Best Practices Checklist by the Canadian Association of Emergency Physicians (CAEP) [38]. Consequently, the RAFF-3 trial [39] sought to investigate the impact of implementing this guidance [39]. It was conducted as a stepped-wedge cluster randomized trial at 11 large community and academic hospital EDs in five Canadian provinces and enrolled consecutive AF/AFL patients. The study intervention was the introduction of the CAEP Checklist with the use of a knowledge translation–implementation approach that included behavior change techniques and organization-/system-level strategies. The RAFF-3 trial led to optimized care of AF/AFL patients with decreased ED lengths of stay by 20.9%, increased ED rhythm control by drug or electricity, and no increase in adverse events [39].

Most published AF treatment protocols have been validated in high-volume tertiary care centers. De Meester et al. demonstrated that the utilization of an AF treatment protocol could reduce admission rates even in a community-level hospital ED, based primarily on rate control rather than rhythm control [21]. Specifically, in this retrospective cohort study, there was a pre-implementation period from March 2013 to February 2014 with 586 patients included and a post-implementation period from March 2015 to February 2016 with 522 patients included. The primary outcome was hospital admissions and indeed these were greatly reduced in the post-implementation group (67.4% vs. 80.4% in the pre-implementation group) [21].

## 6. Long-Term Outcomes

While the short-term (<7-day) safety and efficiency of electrical cardioversion for (ED) patients with AF have been established, the 30-day outcomes with respect to stroke, thromboembolic events, or death were first investigated in a cohort of 1233 patients during the period from 2000 to 2007. There were no deaths, strokes, or other thromboembolic events in the first 30 days following cardioversion [40].

A prospective cohort study was conducted in six academic hospital EDs enrolling patients who had AF/AFL onset within the past 48 h. Patients were followed for 30 days by health record review and telephone. Among the 1091 patients enrolled, 9% were admitted to the hospital and 80.1% were converted to a sinus rhythm. Although 10.5% had adverse events within 30 days, there were no related deaths and only one stroke (0.1%). Patients who left the ED in a sinus rhythm were much less likely to experience an adverse event (*p* < 0.001) [41].

## 7. Ongoing AF Pathways

The U-CARE AF pathway was developed by the University of California to standardize the management of patients presenting to the ED with primary AF [42]. In addition to the management and stabilization of acute AF, the U-CARE AF pathway aims to improve the adherence to anticoagulation and reduce unnecessary hospital admissions whenever safe with a quick outpatient follow-up. The initial goal of the pathway is the control of the ventricular response that is initiated pharmacologically in all patients (desired rate control < 110 bpm). Although current guidelines consider it safe to cardiovert patients who can definitely pinpoint the start of AF within the last 48 h, in the U-CARE AF pathway, a 12 h cutoff is used instead. Regarding chemical cardioversion, the preferred agents are oral propafenone, oral flecainide, and intravenous procainamide. If a patient is not cardioverted, they may be discharged on rate control medication and anticoagulation and the decision of whether cardioversion should be attempted again is delegated to the outpatient clinic.

Contrary to the conservative U-CARE AF pathway, the expert consensus in Canada limits hospital admissions only to highly symptomatic AF patients with decompensated heart failure or myocardial ischemia and to those (highly symptomatic) in whom adequate rate control cannot be achieved. ED management priorities include the assessment for potential hemodynamic instability and careful assessment of the time of AF onset. For stable patients with recent-onset AF or atrial flutter, there are two competing strategies for management, either rate control or rhythm control [43,44,45]. The rate control approach consists of ventricular rate control, oral anticoagulation, no attempt to convert the patient to a sinus rhythm in the ED, and delayed cardioversion after 4 weeks, if indicated. With the rhythm control approach, attempts are made to cardiovert patients to a sinus rhythm in the ED, either pharmacologically or electrically, and then discharge them home in the sinus rhythm [18,46,47].

## 8. HEROMEDICUS Protocol

The HEROMEDICUS protocol was developed by the National and Kapodistrian University of Athens to standardize the management of AF in the ED. Our purpose is not only to reduce hospital admissions in a safe and effective environment but also to ensure the highest percentage achieve a sinus rhythm rate through an aggressive rhythm control strategy. Most importantly, the protocol is designed and executed by expert electrophysiologists in collaboration with cardiologists in the absence of emergency medicine doctors. The study protocol was approved by the Ethics Committee and all included patients provided an informed consent form.

**Figure 1:**
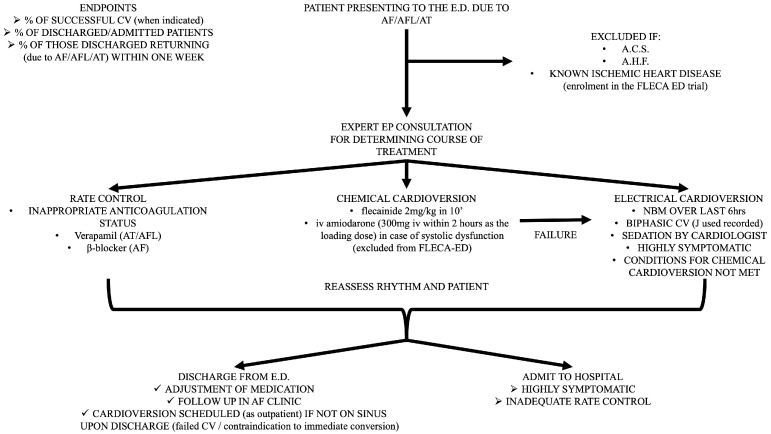
Detailed flowchart presenting all the steps of the HEROMEDICUS protocol for AF management in the ED.

All patients who visit the ED with a primary diagnosis of AF or AFL are included in the study, even if the time from arrhythmia initiation cannot be specified. The only prerequisite is a visit to the ED due to palpitation, shortness of breath, fatigue, or other relative symptoms related to AF, unless the patients had a definite diagnosis of AF and a precise self-report of the AF episode. Patients who present to the ED for other reasons, such as infection, anemia, or stroke, and receive a concomitant diagnosis of AF (either short term or long term) are excluded from the HEROMEDICUS protocol. A standard bedside echo is performed in every patient at the ED in order to provide a rough assessment of the left ventricular systolic function and valvular function. As mentioned above, patients with severe systolic dysfunction (ejection fraction < 40%) and signs of acute heart failure are considered as having acute heart failure as a primary diagnosis and excluded from the protocol (Figure 1).

Patients with ischemic heart disease and a preserved ejection fraction without indications of acute coronary syndrome are randomized in the setting of the FLECA-ED study and excluded from the HEROMEDICUS protocol [48,49]. Meanwhile, patients with ischemic heart disease and severe systolic dysfunction (ejection fraction <40%) without signs and symptoms of acute heart failure are excluded from the FLECA-ED study and excluded from the HEROMEDICUS protocol. Furthermore, patients with indications of acute coronary syndrome are also excluded (Figure 1).

Enrollment started in September 2023 and will end in September 2025. Retrospective data will be collected from the ED archive and hospital data in the periods September 2021–September 2022 and September 2022–September 2023 in order to assess the impact of the COVID-19 pandemic on AF visits and admissions from the ED. Prospectively selected data for between September 2023 and 2025 will be compared to data for this retrospective cohort from between September 2021 and 2023.

A special application has been created by P.T. for the purpose of the HEROMEDICUS protocol and installed in the mobile phones of the cardiologists in charge in the ED. The initial baseline clinical parameters are recorded and inclusion and exclusion criteria are implemented by means of a bedside echocardiogram. Furthermore, an emphasis is placed on the prior history of AF episodes and ED visits and hospital admissions, along with the use of antiarrhythmics and the anticoagulant status (warfarin and time in therapeutic INR range, and type and dose of direct oral anticoagulant).

*Expert electrophysiologic consultation (Figure 1)*

- Decision for rate control and subsequent (>21 days) electrical cardioversion in case of poor anticoagulant status and long (>48 h) AF detection
  ⇒ Use of verapamil in case of AFL or atrial tachycardia;
  ⇒ Use of β-blockers in case of AF.
- Chemical cardioversion if immediate pill in the pocket administration of propafenone or flecainide did not exceed 300 mg or 200 mg, respectively
  ⇒ Use of i.v. flecainide (150 mg i.v. within 10 min);
  ⇒ Use of i.v. amiodarone (300 mg i.v. within 2 h as the loading dose) in case of left ventricular systolic dysfunction without signs or symptoms of acute heart failure, which are excluded from the FLECA-ED study.
- Electrical cardioversion
  ⇒ Nil by mouth for the preceding 6 h;
  ⇒ Sedation and analgesia provided by cardiologist in ED (i.v. use of midazolam and pethidine);
  ⇒ Use of biphasic synchronized cardioversion (registration of delivered energy).

Cardioversions are all performed in the ED by trained cardiologists (transesophageal echo is not performed in any case) and the safety and efficacy of this strategy constitutes a secondary endpoint of the HEROMEDICUS protocol.

*Discharge from ED (Figure 1)*

- Modification of outpatient antiarrhythmic use based on discharge ECG.
- Outpatient follow-up in AF clinic.
- Programmed electrical cardioversion in case of poor anticoagulant status and long (>48 h) AF detection (use of transesophageal echocardiogram only in cases of suspected severe valvulopathy).
- All patients will receive anticoagulants (any type of NOAC in the proper dose according to age, renal function, and weight) based on CHADSVASc score. If the CHADSVASc score is 0 in men and 1 in women, patients receive anticoagulants if the estimated duration of the episode is >12 h.

The preference for i.v. flecainide is based on the largest published network meta-analysis to date, which indirectly compared and ranked antiarrhythmic agents focusing exclusively on adults with paroxysmal AF [50]. Forty-one randomized controlled trials (6013 patients) were included, with i.v. vernakalant and i.v. flecainide found to have the highest conversion rate within 4 h, possibly allowing discharge from the ED and reducing hospital admissions [50]. Vernakalant was not selected due to its prohibitive cost in the setting of the ED.

## 9. Initial Results

During the first 100 days of the study period, 63 patients visited the ED with a primary diagnosis of AF (*n* = 55) or AFL (*n* = 8) and were included in this study. It is notable that in half of them, this was the first episode of AF (*n* = 32). Among the 31 patients with a known history of AF, 5 had undergone previous AF ablation. Electrical cardioversion was performed in 18 patients and restoration of SR was achieved in 17 of them. Ultimately, only two patients were admitted to the hospital (3.2%) and both of them were discharged within 48 h.

## 10. Discussion

The incidence and prevalence of AF continue to increase worldwide, in accordance with the increasing burden of related comorbidities [2,3,4,5,6,7]. Subsequently, AF is becoming a very common reason for ED visits, now accounting for 0.5% of presenting reasons [11,12,13,14]. Registry data have indicated that around 60% of AF patients who present to the ED are admitted, leading to an increase in AF-related hospital admissions [11,12,13,14]. The above have pointed out the need for strategies for the safe and effective management of AF in the ED in order to avoid admissions and reduce costs [11,12,13,14].

Management of AF in the setting of the ED varies between countries and healthcare systems. Several trials of AF rhythm control strategies, including cardioversion, have demonstrated that AF in the ED can be treated safely and effectively, avoiding admission [11,12,13,14,15,16,17,18,19,20,21,22,23,24,25,26]. It is noteworthy that rate control rather than rhythm control is historically the preferred strategy in the United States, while in other Western countries such as Canada, Australia, and the United Kingdom, rhythm control is attempted in patients with AF who visit the ED [26,41,51]. The most plausible reason to justify a conservative rather than an aggressive strategy in the management of AF is the absence of specific guidelines from diverse societies. In this direction, in the multicenter, randomized, open-label, noninferiority RACE 7 ACWAS trial, a wait-and-see approach was found to be noninferior to early cardioversion in achieving a return to a sinus rhythm at 4 weeks in patients presenting to the ED with recent-onset, symptomatic AF [52]. It should also be taken into account that in certain hospitals, there is a lack of ED resources to perform electrical cardioversion, as well as a lack of payer scrutiny of short hospitalizations or readmissions due to AF, in contrast to other conditions such as angina, syncope, and decompensated heart failure [52]. On the other hand, the very encouraging results yielded by aggressive AF management protocols, resulting in significantly fewer hospitalizations without evidence of worse outcomes, along with data suggesting a patient preference for sinus rhythm restoration, constitute the fundamental reasons for pursuing an early cardioversion strategy [53,54,55,56,57].

## 11. Conclusions

In the present study, we have described the epidemiology and characteristics of AF patients presenting to the ED, depicting the continuous increase in AF-related ED visits and hospital admissions. We have also presented the differences in the management of AF in the setting of the ED between healthcare systems and the tremendous impact of these diverse management strategies on AF-related hospital admissions. Lastly, we have analyzed the design of the HEROMEDICUS protocol, which constitutes an electrophysiology-based aggressive rhythm control strategy in patients with AF in the ED setting, and offered initial data on its application.

## Figures and Tables

**Table 1:** Temporal trends in AF-related ED visits.

Study	ED Visits	AF ED Visits	AF ED Visits/Year	Increase in AF ED Visits	Study Period
McDonald AJ et al. [14]		2,700,000	300,000 (1993) 564,000 (2004)	88%	1993–2004
Lin M et al. [13]			434,382 (2006) 537,801 (2014)	30.9%	2006–2011
Rozen G et al. [11]	233,007,973	3,886,520	411,406 (2007) 537,801 (2014)	30.7%	2007–2014

**Table 2:** Temporal trends in AF-related admission rates.

Study	AF ED Visits	AF Admissions	AF Admission Rate	Study Period
McDonald AJ et al. [14]	2,700,000		64%	1993–2004
Lin M et al. [13]	434,382 in 2006 568,561 in 2011		69.7% in 2006 67.4% in 2011	2006–2011
Rozen G et al. [11]	3,886,520	288,225 in 2007 333,570 in 2014	70% 2007–2010 62% in 2014	2007–2014

**Table 3:** Trials on different strategies for AF management in the ED.

Study	AF Duration	Intervention	Study Type	Hospital Type	Number of Patients	SR Rate (%)	Admission Rate (%)	Adverse Events Rate (%)	ED Return Rate (%)
Burton [18]	<48 h	DCCV	Retrospective cohort	Tertiary	388	86	14	8	10 (10 d)
Stiell [20]	Acute onset	PROC iv	Retrospective cohort	Tertiary	341	52(AF), 28(AFL)	5.6	10	2.9 (7 d)
Stiell [26]	Recent onset	PROC iv +/− DCCV	Retrospective cohort	Tertiary	600 (PROC), 243 (DCCV)	58 (PROC) 92 (DCCV)	3.2	7.6	8.6 (7 d)
Scheuermeyer [23]	<48 h	PROC iv +/− DCCV or DCCV +/− PROC	Multicenter randomized study	Tertiary care	41 PROC 43 DCCV	100 PROC 98 DCCV	0	25	12.2 (3 d) 2.2 (30 d)
Martin [24]	Recent onset	CC or DCCV	Multicenter observational cross-sectional	Tertiary care, community	421	70	14	n/a	n/a
Ptaszek [25]	New or recurrent	CC or DCCV	Prospective two-stage study at two hospitals	Tertiary care, community	104 routine care 104 AF pathway	61 routine care 76 AF pathway	55 routine care 15 AF pathway	n/a	11 (in 4 months)
De Meester [21]	New or recurrent	DCCV if rate control failed	Retrospective cohort	Community	1108	n/a	67	n/a	1 (3 d) 3.6 (30 d)

CC: chemical cardioversion, DCCV: direct current cardioversion, PROC: procainamide.

## Data Availability

Data is unavailable due to privacy.
